# Gaze and Eye-Tracking Perspectives for Psychological Research: A Narrative Review of Advances From 2012 to 2025

**DOI:** 10.7759/cureus.104753

**Published:** 2026-03-06

**Authors:** Maria Laura Mele, Stefano Federici

**Affiliations:** 1 Psychology, Myèsis Insight Center, Center for Research and Psychotherapy, Rome, ITA; 2 Psychology, Department of Philosophy, Social &amp; Human Sciences and Education, University of Perugia, Perugia, ITA

**Keywords:** cognitive and affective processes, eye tracking, gaze analysis, human–computer interaction, psychological assessment

## Abstract

Over the past 14 years, eye-tracking technology has emerged as a transformative tool in psychological research, offering insights into cognitive processes, emotional responses, and behavioral patterns. This narrative review synthesizes advancements in eye-tracking applications from 2012 to 2025 across four primary psychological domains: (i) cognitive psychology, (ii) emotional and affective research, (iii) psychological variables in human-computer interaction, and (iv) clinical psychological assessments. Using selected Preferred Reporting Items for Systematic Reviews and Meta-Analyses (PRISMA) 2020-informed reporting items to improve transparency, the structured narrative synthesis discusses 70 peer-reviewed publications. The research underscores methodological and theoretical progress, including the integration of multimodal measurements and the advent of machine learning approaches for gaze data analysis. The thematic synthesis indicates that eye-tracking measures have been explored as potential indicators in clinical research contexts and as tools for examining cognitive workload through pupillary responses. However, considerable methodological limitations persist, including discrepancies in technical accuracy, calibration drift, and the requirement for uniform methods among studies. This study emphasizes that future investigations should prioritize transparent reporting and ethical safeguards regarding gaze data privacy and informed consent.

## Introduction and background

The eye-mind hypothesis, which asserts that eye movements reflect cognitive processes, is a foundational principle in cognitive science [1]. For over 50 years, this methodology has driven research that establishes eye tracking as a technique for analyzing information processing in real time [1]. Eye tracking refers to the recording and analysis of eye movements, including fixations (periods during which gaze remains relatively stable), saccades (rapid eye movements between fixation points), and pupillary responses (changes in pupil diameter associated with cognitive and emotional processes) [1]. The domain has evolved from its early applications in reading studies to encompass complex visual and cognitive tasks, thus positioning eye tracking as a key element of psychological research [2].

Fourteen years ago, the authors (M.L.M. and S.F.) published a narrative review outlining the state of eye-tracking technology and its applications within psychological science [1]. Since then, substantial technological advances have reshaped the field, including improvements in hardware accuracy, data processing, and analytical approaches [3-5]. Eye-tracking methodologies have progressively expanded across multiple domains, notably psychophysical research [6] and clinical psychological assessment [2]. These developments call for an updated and comprehensive synthesis to clarify the current state of the discipline and to delineate emerging research trajectories.

This narrative review aims to synthesize advancements in eye-tracking applications within psychological research from 2012 to 2025. While systematic reviews follow specific methodologies to answer specific questions and scoping reviews map broad areas, narrative reviews offer a comprehensive overview of a topic, integrating diverse perspectives and emerging trends. The work investigates four primary psychological domains: (i) cognitive psychology applications, (ii) emotional and affective research, (iii) psychological variables in human-computer interaction, and (iv) clinical psychological evaluations. This study examines theoretical and methodological developments in each discipline, emphasizing the growth of the field and identifying emerging prospects and future research objectives. It is important to note that while eye-tracking measures offer promising exploratory insights in clinical contexts, their applications remain largely investigational and are not yet established for routine diagnostic or decision-making purposes.

Building upon our previous work [1], this review provides a critical update by capturing the shift toward mobile and wearable eye-tracking solutions, multimodal integration, and machine learning-based analyses that have emerged between 2012 and 2025, advancements that were not addressed in earlier syntheses.

## Review

Method

This narrative review was conducted following Preferred Reporting Items for Systematic Reviews and Meta-Analyses (PRISMA) 2020-informed reporting practices to enhance transparency and methodological clarity. Selected PRISMA 2020 [7] transparency components were applied, including a structured search strategy, predefined inclusion and exclusion criteria, duplicate removal procedures, and a PRISMA-informed flow diagram. However, this review does not constitute a full systematic review, as no protocol registration or formal risk-of-bias assessment was conducted. The primary research question was: What significant methodological and theoretical advancements characterized eye-tracking applications in psychology between 2012 and 2025?

Literature searches were performed across five databases (Scopus, PubMed, Web of Science, PsycINFO via EBSCO, and IEEE Electronic Library) on January 16th, 2026. A preliminary thematic exploration was used to inform the development of a structured search query that incorporated proximity operators (W/3 for within-three-words proximity), Boolean logic, critical technological terminology (e.g., “eye-tracking,” “gaze,” “wearable eye tracker”), and pertinent application domains (“psychology,” “cognitive processes,” “human-computer interaction,” “usability”), date (2012-2025), document type (peer-reviewed articles and systematic reviews), and subject area (psychology, neuroscience, computer science) (Table 1).

**Table 1:** Bibliographic research query

RESEARCH QUERY
(TITLE-ABS-KEY(“eye-tracking” W/3 (“visual attention” OR “cognitive load” OR “emotion recognition”)) OR TITLE-ABS-KEY(“gaze” W/3 (“psychology” OR “cognitive” OR “behavioral”)) OR TITLE-ABS-KEY(“wearable eye tracker”) OR TITLE-ABS-KEY(“webcam eye tracking”) OR TITLE-ABS-KEY(“mobile eye tracking”) OR TITLE-ABS-KEY(“desktop eye tracker”)) AND TITLE-ABS-KEY(“human-computer interaction” OR HCI OR “usability” OR “user experience” OR “neuroergonomics”) AND (PUBYEAR > 2011 AND PUBYEAR < 2026) AND DOCTYPE(ar OR re) AND SUBJAREA(psyc OR neur OR comp) AND NOT TITLE-ABS-KEY(“non-human” OR animal OR “conference proceeding” OR “book chapter” OR “in press”)

The inclusion and exclusion criteria are detailed in Table 2.

**Table 2:** Inclusion and exclusion criteria

Inclusion criteria	Exclusion criteria
Peer-reviewed original research articles or systematic reviews published in indexed journals	Studies not involving human subjects
Research conducted from January 2012 to January 2026	Gray literature and nonpeer-reviewed sources
Studies in the domains of Psychology, Neuroscience, or Computer Science	Research focused exclusively on pharmacological treatments without a psychotherapy component
Employment of eye-tracking technologies (desktop-mounted systems, head-mounted wearable devices, webcam-based remote tracking)	Clinical case reports
Empirical data obtained from human participants (experimental, observational, or correlational designs)	Single-case studies or anecdotal reports, unless they contributed to a meta-analysis or systematic review
	Research focused exclusively on technological development without a deliberate discussion of psychological implication of the interaction

**Figure 1:**
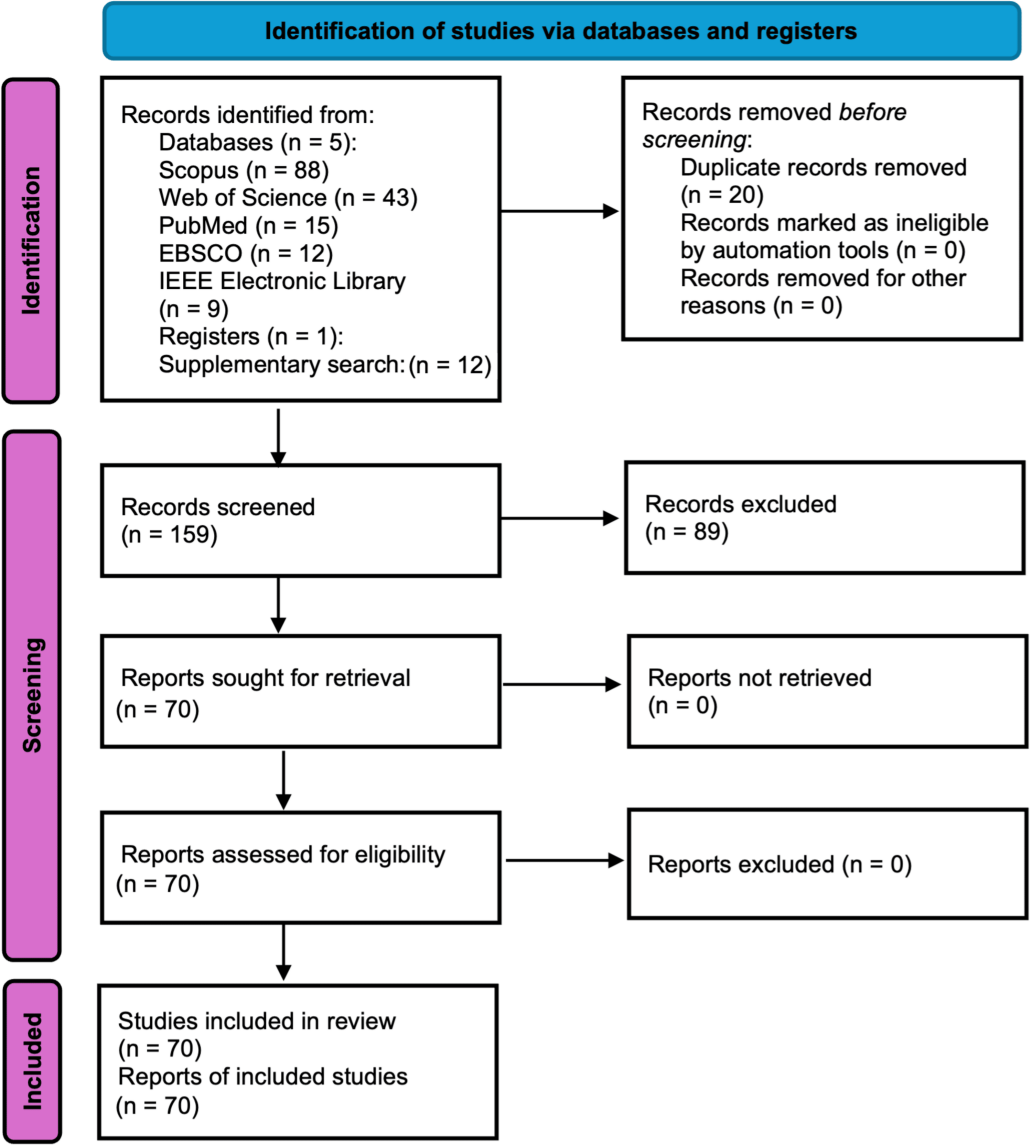
PRISMA-informed flow diagram illustrating the study selection process This diagram is provided to enhance transparency of the narrative review process [7]; PRISMA: Preferred Reporting Items for Systematic Reviews and Meta-Analyses.

The structured literature search yielded 179 records in total: 167 from five databases-Scopus (n = 88), Web of Science (n = 43), PubMed (n = 15), EBSCO (n = 12), and IEEE Electronic Library (n = 9)-and 12 additional records identified through a targeted supplementary search in PubMed, PubMed Central, and PsycINFO (January 16, 2026), all of which met the inclusion criteria (Figure 1). Duplicate removal was achieved through a blend of automated techniques (Zotero version 7.0.0, utilizing DOIs, titles, and author comparisons) and manual validation, leading to the discovery of 20 duplicates (17 identified automatically, 3 manually) from the 179 initially retrieved. Studies were prioritized for discussion based on their methodological innovation and theoretical relevance to the four identified domains. To minimize selection bias, the inclusion criteria were applied consistently by one reviewer (M.L.M.) and verified by a second (S.F.), ensuring that the narrative focus remained on high-impact advancements within the 2012-2025 timeframe. Any discrepancies between the reviewers during study selection were resolved through discussion and consensus. No unresolved disagreements remained after this process. A total of 159 unique records were forwarded for screening.

After the application of predefined inclusion and exclusion criteria, a manual refinement step was conducted to remove 89 records. The final dataset comprised 70 research studies classified into four psychological macro-areas by extensive content analysis (Figure 1). However, only the most representative and methodologically significant were recognized individually, in accordance with journal editorial requirements regarding reference selection and reporting. Studies were prioritized for discussion based on explicit criteria including their methodological innovation and robustness, and theoretical relevance to the psychological domain, ensuring a focus on high-impact advancements within the specified timeframe. This selective emphasis was intended to support thematic synthesis and conceptual clarity rather than exhaustive coverage of all eligible studies. A complete list of all selected studies that were not discussed in detail in the narrative review is provided in Appendix A. The data extraction encompassed study characteristics, eye-tracking methods, psychological constructs, primary findings, and methodological limitations.

In line with the narrative nature of this synthesis, a formal risk-of-bias assessment of the included studies was not conducted, representing an inherent limitation of this narrative review format. However, study quality was informally considered during the selection process through qualitative factors such as publication in peer-reviewed and indexed journals, and the clarity and rigor of their reported methodologies. This informal consideration aimed to ensure the inclusion of relevant research.

Results

The literature search yielded a diverse corpus of research, from which 70 studies were selected to support a narrative synthesis of key thematic and methodological developments. The following sections provide a thematic overview of advancements rather than an exhaustive systematic tabulation of all available evidence. The final dataset included 70 studies divided into four psychological domains: (i) psychological factors in human-computer interaction (HCI) (n=25, 35.7%), (ii) cognitive psychology applications (n=20, 28.6%), (iii) clinical psychological assessments (n=18, 25.7%), and (iv) emotional and affective research (n=7, 10.0%). The distribution of these studies across domains is summarized in Figure 1 and Table 2, while a comprehensive list of all 70 identified records is provided in Appendix A to ensure transparency of the selection process.

The first category, psychological factors in HCI (psychological domain 1), represented the predominant segment, encompassing studies into cognitive load, visual attention allocation, information processing techniques, and perceptual systems in interface design and usability, as demonstrated by foundational HCI eye-tracking research studies, e.g., [8-10]. These studies emphasized eye-tracking methodologies grounded in psychological theoretical frameworks, extending beyond purely descriptive usability metrics. While HCI represents a substantial portion of recent eye-tracking literature, reflecting rapid technological development, comparable conceptual attention is given to cognitive, emotional, and clinical domains to provide an integrated psychological perspective.

The applications of cognitive psychology (psychological domain 2) included research on key cognitive processes such as attention, memory, perception, executive function, and visual search, in which recognized theoretical and empirical frameworks were employed. This field predominantly used eye tracking as the primary instrument to examine the temporal and spatial dynamics of cognitive processing, e.g., [11,12].

Clinical psychological research (psychological domain 3) included studies employing eye tracking to assess and characterize visual attention and oculomotor patterns associated with psychological and psychiatric conditions, such as attention-deficit/hyperactivity disorder (ADHD), autism spectrum disorder (ASD), schizophrenia, and mood disorders [2]. Studies examining atypical visual attention and oculomotor behavior suggest that eye-tracking measures may offer exploratory insights as complementary indicators in clinical psychological research [2,6]. However, these findings should be interpreted as exploratory and investigational.

Finally, emotional and affective research (psychological domain 4) included studies that investigated emotional states, affective responses, and emotion recognition through eye-tracking techniques. This research domain encompassed studies investigating affective-related gaze patterns and, where applicable, pupillary responses during emotional processing, e.g., [13,14]. Although numerically smaller, this body of work contributes to understanding affective modulation of attention and autonomic responses.

To further enhance transparency and facilitate comparative analysis, a summary table (Appendix B) has been added, outlining key characteristics of the included studies such as psychological domain, sample type, primary methodology, and main findings.

Domain 1: Psychological Factors in Human-Computer Interaction (HCI)

Eye tracking is a key tool in clarifying HCI by depicting the allocation of visual attention by users when interacting with interfaces [8,9]. Studies in this domain are consistent in showing that gaze patterns signal cognitive stress during task performance [15,16]. Practitioners have used fixation duration, saccade amplitude, and pupil diameter to assess attentional requirements across different interface types, including traditional desktop applications, mobile platforms, and immersive environments [10].

A recent methodological improvement in this field involves the integration of eye tracking with supplementary psychophysiological measures (e.g., electroencephalography, heart rate variability) to produce multimodal assessments of user experience [16]. This integration enhances validity by incorporating complementary aspects of cognitive and emotional engagement. Furthermore, machine learning techniques have evolved for the automatic classification of user states (e.g., confusion, engagement, frustration) based on gaze patterns, indicating potential applications in adaptive interface design [4,17].

The principal findings demonstrate that fixation patterns and saccade characteristics reliably predict task difficulty and user competence [9]. Inexperienced users typically exhibit extended fixation durations and increased saccade frequency compared to professionals, thus suggesting greater cognitive processing demands [18]. Furthermore, research suggests that gaze-contingent interfaces, which adjust according to user gaze, may enhance usability and reduce cognitive load, particularly in complex information environments [19].

Domain 2: Cognitive Psychology Applications

Eye tracking has improved the understanding of fundamental cognitive processes considerably by providing real-time, objective measurements of attention and information processing. Research on attention reveals that fixation patterns reflect the locus of attentional focus, while saccade latency and amplitude reflect attentional shifting and disengagement [12]. Studies on visual search consistently show that eye tracking uncovers the systematic exploration strategies employed by individuals to identify targets, with patterns varying according to target salience, expertise, and task demands [11].

Research utilizing eye tracking in memory studies has revealed that gaze patterns during encoding predict later memory performance. Extended fixation durations on previously examined items correlate with improved recognition memory, and eye tracking during retrieval reveals systematic search patterns that reflect memory organization [6,11]. Moreover, research on reading shows that eye movements reveal the immediate cognitive processes engaged in language comprehension, with fixation patterns affected by word frequency, predictability, and grammatical complexity [11].

Investigations on executive function via eye tracking have revealed that gaze patterns reflect cognitive control mechanisms, particularly in tasks requiring inhibition or task switching, while studies on antisaccade tasks, which require the inhibition of reflexive saccades toward peripheral stimuli, reveal that gaze patterns reflect the engagement of inhibitory control mechanisms [12]. Furthermore, eye tracking has demonstrated the importance of attention in working memory, suggesting that gaze patterns during memory retention tests reflect the active rehearsal of items to be recalled [11].

Current evidence suggests that eye-tracking measures could serve as potential indicators in clinical settings. However, given the narrative nature of this synthesis, these findings should be interpreted as preliminary trends rather than established clinical biomarkers.

Domain 3: Clinical Psychological Assessments

Eye tracking, which provides quantitative measures of attention and perceptual processes, has been increasingly explored as a complementary exploratory tool for clinical psychological evaluation [2]. However, it is important to highlight that their applications remain investigational and are not yet suitable for routine clinical use or independent diagnostic decision-making.

Investigations into ADHD employing eye-tracking techniques indicate atypical patterns of sustained attention and impulse control, with individuals diagnosed with ADHD exhibiting increased saccade frequency and reduced fixation stability compared to control groups [13]. These findings suggest a potential role for eye-tracking measures as complementary indicators in clinical assessment; however, substantial methodological heterogeneity and limited standardization across studies currently constrain their interpretability [2,14].

Investigations into ASD utilizing eye-tracking technology have revealed distinctive gaze patterns, particularly reduced attention to social stimuli (e.g., faces, biological motion) and an increased focus on nonsocial objects [13]. Atypical gaze patterns have been shown to characterize specific populations and psychological profiles, suggesting their potential relevance for developmental research and the monitoring of cognitive and behavioral changes over time [2,13]. However, the heterogeneity in gaze patterns across individuals with ASD indicates that eye tracking should be incorporated into a thorough assessment battery rather than used as a standalone diagnostic tool [2,13].

Research on schizophrenia and mood disorders has revealed alterations in smooth pursuit eye movements and saccadic control, with some findings suggesting potential biomarker applications [6]. The clinical applicability of these findings is limited by the small sample numbers, a lack of established methodologies, and insufficient data about diagnostic sensitivity and specificity, and future research should prioritize comprehensive, longitudinal studies employing standardized eye-tracking methodologies and rigorous validation against established diagnostic standards.

Domain 4: Emotional and Affective Research

Eye tracking has shown consistent associations between gaze patterns and emotional states. Pupillary responses, particularly changes in pupil diameter, serve as a reliable measure of emotional arousal, with pupil dilation correlating with emotional intensity in response to both positive and negative stimuli [14]. Furthermore, gaze patterns in emotion perception reveal that individuals preferentially concentrate on emotionally salient features of stimuli, with attentional patterns varying according to individual differences in emotional reactivity and emotion regulation skills [13].

Research on facial expression recognition reveals that gaze patterns during face observation demonstrate methodical exploration techniques, with individuals predominantly concentrating on the eyes and mouth, which provide emotion-related information [13]. Unusual gaze patterns when observing faces have been documented in clinical populations, including individuals with ASD and social anxiety disorder, suggesting potential therapeutic relevance [13]. Moreover, studies examining gaze patterns while watching emotional movies reveal that attention allocation is associated with emotional engagement and may predict emotional memory [14].

Machine learning methods have been used on gaze data to classify emotional states with significant precision. These approaches employ several gaze attributes (fixation duration, saccade amplitude, pupil diameter) to develop predictive models of emotional states [14]. Marked individual variations in gaze patterns during emotional processing lead to the need for customized calibration for enhanced categorization accuracy [13].

Discussion

The thematic synthesis across the four psychological domains shows a progression in the methodological democratization of technology, enabled by the emergence of affordable, accessible equipment such as webcam-based eye trackers [4,20,21]. This has expanded applicability across diverse application sectors, including education, healthcare practice, and consumer research [10,15]; however, the resulting accessibility highlights methodological challenges, such as the fact that lower-cost systems often demonstrate reduced spatial accuracy and temporal resolution compared to laboratory-grade instruments [22]. Future research should report minimum technical parameters (such as spatial accuracy, sampling rate, and calibration procedures) to enhance reproducibility, particularly when low-cost or webcam-based systems are employed. The resulting proliferation of different hardware with distinct technical specifications makes cross-study comparisons and meta-analytic synthesis difficult to achieve, underscoring the need for standardized protocols and transparent reporting [2].

Pupil diameter has been widely used as a sensitive physiological measure associated with variations in cognitive workload across different task conditions [2,14]. Pupil dilation correlates with task complexity, cognitive exertion, and attentional demands, making it a key measure in both basic cognitive research and practical applications like driving or interface design [8,16]. Its application includes clinical settings, where altered pupillary reactivity has been noted in conditions such as ADHD and depression [6,13], suggesting potential relevance for clinical research. From a research perspective, this generates opportunities for real-time adaptive systems that can adjust task difficulty or provide assistance based on a user’s evaluated cognitive load [11,15], with implications for education and clinical rehabilitation.

A separate tendency to enhance construct validity through the multimodal integration of eye tracking and additional psychophysiological data has emerged [2,19]. The combination of gaze data with electroencephalography, electrocardiography, or facial expression analysis provides a comprehensive assessment of cognitive and emotional processes, thereby minimizing dependence on a single parameter [13,14]. This paradigm has emerged as a promising approach in clinical research, as evidence from different markers can enhance the interpretability and robustness of assessment outcomes [6,9]. Furthermore, it enables the investigation of interconnections across diverse physiological systems, hence improving the theoretical understanding of integrated cognitive-emotional processing [8,11].

Alongside hardware and multimodal advancements, machine learning has become a new tool for extracting meaningful patterns from complex gaze data [8,9,15,23]. Supervised learning algorithms are increasingly utilized to categorize user states (e.g., engagement), emotional responses, and clinical scenarios with an accuracy that may outperform traditional approaches in specific task settings [13,14]. Unsupervised machine learning approaches have demonstrated their significance in identifying new gaze patterns and user strategies without pre-established classifications [8,23]. Despite their advantages, these applications could face challenges, including the risk of overfitting on limited datasets and the constrained interpretability of complex models, leading to the need for rigorous validation and transparent reporting [2]. While the use of eye tracking in basic cognitive tasks is well-established, its application in machine learning-based diagnostics and real-time clinical monitoring remains an emerging and, in some respects, speculative field that requires external validation on independent datasets and assessment of generalizability across populations before practical implementation can be considered.

Notwithstanding these achievements, significant methodological and technological limitations persist. Calibration drift is a prevalent issue, particularly in prolonged or naturalistic studies [20]. Variability arising from individual differences in ocular anatomy and gaze patterns necessitates customized calibration [22]. Moreover, while wearable eye trackers enable the collection of real-world data, they reduce accuracy and increase noise levels [15,21]. Progressing the field requires research to prioritize established protocols for study design and analysis [2], and large-scale studies to confirm normative patterns [9,11].

Emerging technologies offer promising prospects for overcoming limitations. These perspectives should be interpreted as forward-looking research directions rather than near-term clinical implementations. High-speed systems exhibit rapid cognitive processes [22], whilst virtual reality and augmented reality environments integrated with eye tracking facilitate immersive, ecologically valid research [6,10,19]. Neuroimaging integration would enhance the understanding of the brain correlates associated with attention and gaze [2,8], whilst the validation of potential biomarkers against diagnostic criteria could benefit clinical applications [6,13].

Overall, the complexity of gaze data processing could raise ethical concerns. Gaze patterns may reveal sensitive personal information, creating the potential for exploitation [9,14]. Ethical frameworks must include data governance and informed consent to ensure responsible implementation [15,19].

## Conclusions

This paper outlines advancements in eye-tracking applications in psychological research from 2012 to 2025. Technological democratization has enhanced accessibility, whereas multimodal integration and machine learning have augmented analytical complexity. Eye tracking has proven valuable for investigating cognitive processes characterizing emotional and affective responses, and advancing research on human-computer interaction, while offering exploratory insights into potential clinical applications. Nonetheless, methodological heterogeneity limits cross-study comparisons. A central methodological insight emerging from this review is that technological innovation must be accompanied by transparent reporting standards to ensure interpretability and comparability across studies. Advancing the field requires rigorous protocols and ethical safeguards. The prospective combination of neuroimaging, artificial intelligence, and immersive environments may expand opportunities.

Overall, the 2012-2025 period marks a transition from laboratory-bound eye tracking to pervasive, AI-driven gaze analysis. This review underscores the importance of balancing technological innovation with methodological rigor and ethical responsibility to support integrative psychological models and future research development.

## Appendices

Appendix A

**Table 3:** List of all the studies reviewed

S. No.	Author (Year)	Article title	Journal abbreviation	DOI/URL
1	Akinyelu AA, Blignaut P (2021)	Convolutional Neural Network-Based Technique for Gaze Estimation on Mobile Devices	Front Artif Intell	10.3389/frai.2021.796825
2	Alam L et al. (2021)	Active Vision-Based Attention Monitoring System for Non-Distracted Driving	IEEE Access	10.1109/access.2021.3058205
3	Ballenghein U et al. (2019)	Cognitive engagement in emotional text reading: concurrent recordings of eye movements and head motion	Cogn Emot	10.1080/02699931.2019.1574718
4	Ban G, Park W (2024)	Effects of In-Vehicle Touchscreen Location on Driver Task Performance, Eye Gaze Behavior, and Workload During Conditionally Automated Driving: Nondriving-Related Task and Take-Over	Hum Factors	10.1177/00187208241226838
5	Castilla D et al. (2023)	Improving the understanding of web user behaviors through machine learning analysis of eye-tracking data	User Model User Adapt Interact	10.1007/s11257-023-09373-y
6	Chauvin C et al. (2020)	Analyzing the take-over performance in an automated vehicle in terms of cognitive control modes	Trav Hum	10.3917/th.834.0379
7	Chen J et al. (2023)	Effects of Anthropomorphic Design Cues of Chatbots on Users’ Perception and Visual Behaviors	Int J Hum Comput Interact	10.1080/10447318.2023.2193514
8	Chiquet S et al. (2020)	Eye movements to absent objects during mental imagery and visual memory in immersive virtual reality	Virtual Real	10.1007/s10055-020-00478-y
9	Cuve HC et al. (2022)	Validation of Gazepoint low-cost eye-tracking and psychophysiology bundle	Behav Res Methods	10.3758/s13428-021-01654-x
10	Doherty S, O’Brien S (2013)	Assessing the Usability of Raw Machine Translated Output: A User-Centered Study Using Eye Tracking	Int J Hum Comput Interact	10.1080/10447318.2013.802199
11	Friedrich M et al. (2021)	The influence of training level on manual flight in connection to performance, scan pattern, and task load	Cogn Technol Work	10.1007/s10111-020-00663-8
12	Gauselmann P et al. (2023)	Cognitive offloading benefits eye gaze interaction	Appl Cogn Psychol	10.1002/acp.4098
13	Geller J et al. (2020)	GazeR: A Package for Processing Gaze Position and Pupil Size Data	Behav Res Methods	10.3758/s13428-020-01374-8
14	Guerberof Arenas A et al. (2021)	The impact of translation modality on user experience: an eye-tracking study of the Microsoft Word user interface	Mach Transl	10.1007/s10590-021-09267-z
15	Hoogerbrugge AJ et al. (2025)	When is enough enough? Empirical guidelines to determine participant sample size for scene viewing studies	Behav Res Methods	10.3758/s13428-025-02754-8
16	Hu L et al. (2024)	The cognitive experience difference of traditional painting exhibitions with multimedia interventions: evidence from eye-tracking experiment	Curr Psychol	10.1007/s12144-024-07088-w
17	İşbilir E et al. (2019)	Towards a Multimodal Model of Cognitive Workload Through Synchronous Optical Brain Imaging and Eye Tracking Measures	Front Hum Neurosci	10.3389/fnhum.2019.00375
18	Jang Y-M et al. (2013)	Identification of human implicit visual search intention based on eye movement and pupillary analysis	User Model User Adapt Interact	10.1007/s11257-013-9142-7
19	Jia S (2023)	Multi-modal Human-Computer Virtual Fusion Interaction In Mixed Reality	J Appl Sci Eng	10.6180/jase.202311_26(11).0010
20	Kaspere R et al. (2023)	Is machine translation a dim technology for its users? An eye tracking study	Front Psychol	10.3389/fpsyg.2023.1076379
21	Kim S et al. (2024)	How manoeuvre information via auditory (spatial and beep) and visual UI can enhance trust and acceptance in automated driving	Transp Res Part F Traffic Psychol Behav	10.1016/j.trf.2023.11.007
22	Kos’myna N, Tarpin-Bernard F (2013)	Evaluation and Comparison of a Multimodal Combination of BCI Paradigms and Eye Tracking With Affordable Consumer-Grade Hardware in a Gaming Context	IEEE Trans Comput Intell AI Games	10.1109/tciaig.2012.2230003
23	Ladouce S et al. (2022)	Capturing Cognitive Events Embedded in the Real World Using Mobile Electroencephalography and Eye-Tracking	J Cogn Neurosci	10.1162/jocn_a_01903
24	Lanini-Maggi S et al. (2021)	Assessing how visual search entropy and engagement predict performance in a multiple-objects tracking air traffic control task	Comput Hum Behav Rep	10.1016/j.chbr.2021.100127
25	Lee J et al. (2018)	Investigating the correspondence between driver head position and glance location	PeerJ Comput Sci	10.7717/peerj-cs.146
26	Li H et al. (2020)	Think before you speak: An investigation of eye activity patterns during conversations using eyewear	Int J Hum Comput Stud	10.1016/j.ijhcs.2020.102468
27	Li J et al. (2022)	Evaluating the Performance of Machine Learning Algorithms in Gaze Gesture Recognition Systems	IEEE Access	10.1109/access.2021.3136153
28	Li W et al. (2019)	Training a Camera to Perform Long-Distance Eye Tracking by Another Eye-Tracker	IEEE Access	10.1109/access.2019.2949150
29	Lim S, Lee D (2017)	Real-Time Eye Tracking Using IR Stereo Camera for Indoor and Outdoor Environments	KSII Trans Internet Inf Syst	10.3837/tiis.2017.08.012
30	Liu H et al. (2025)	Operational performance, cognitive load, visual attention, and usability of fixed-, manual-, and autonomous-camera control in single- and multiple-camera telemanipulation systems	Appl Ergon	10.1016/j.apergo.2025.104647
31	Lugtenberg G et al. (2025)	Effects of Eye Vergence and Accommodation on Interactions With Content on an AR Magic-Lens Display and its Surroundings	IEEE Trans Vis Comput Graph	10.1109/TVCG.2024.3403261
32	Malak C, Yildirim F (2025)	Pupillary responses to masked and gaze-averted faces	Front Psychol	10.3389/fpsyg.2025.1586186
33	Matulewski J et al. (2022)	Learnability evaluation of the markup language for designing applications controlled by gaze	Int J Hum Comput Stud	10.1016/j.ijhcs.2022.102863
34	Modi N, Kumar Y (2025)	Advancements in Eye Tracking for Visual Attention Analysis Across E-commerce Screen Sizes	Procedia Comput Sci	10.1016/j.procs.2025.04.567
35	Modi N, Singh J (2024)	An analysis of perfume packaging designs on consumer’s cognitive and emotional behavior using eye gaze tracking	Multimed Tools Appl	10.1007/s11042-024-18715-w
36	Molina AI et al. (2014)	Assessing the effectiveness of new devices for accessing learning materials: An empirical analysis based on eye tracking and learner subjective perception	Comput Hum Behav	10.1016/j.chb.2013.04.022
37	Novak D et al. (2013)	Predicting targets of human reaching motions using different sensing technologies	IEEE Trans Biomed Eng	10.1109/TBME.2013.2262455
38	Novák JŠ et al. (2023)	Eye Tracking, Usability, and User Experience: A Systematic Review	Int J Hum Comput Interact	10.1080/10447318.2023.2221600
39	Oliveira L, Carvalho M (2017)	Emotional Design in Web Interfaces	Observatorio (OBS*)	10.15847/obsOBS1122017905
40	Otoo N et al. (2025)	Visceral Notices and Privacy Mechanisms for Eye Tracking in Augmented Reality	IEEE Trans Vis Comput Graph	10.1109/TVCG.2025.3616837
41	Palcu J et al. (2017)	Judgments at Gaze Value: Gaze Cuing in Banner Advertisements, Its Effect on Attention Allocation and Product Judgments	Front Psychol	10.3389/fpsyg.2017.00881
42	Paravati G, Gatteschi V (2015)	Human-Computer Interaction in Smart Environments	Sensors	10.3390/s150819487
43	Proudfoot JG et al. (2016)	More Than Meets the Eye: How Oculometric Behaviors Evolve Over the Course of Automated Deception Detection Interactions	J Manag Inf Syst	10.1080/07421222.2016.1205929
44	Roth SP et al. (2013)	Location matters, especially for non-salient features–An eye-tracking study on the effects of web object placement on different types of websites	Int J Hum Comput Stud	10.1016/j.ijhcs.2012.09.001
45	Ruf T, Ploetzner R (2014)	One click away is too far! How the presentation of cognitive learning aids influences their use in multimedia learning environments	Comput Hum Behav	10.1016/j.chb.2014.06.002
46	Ryu J et al. (2019)	EOG-based eye tracking protocol using baseline drift removal algorithm for long-term eye movement detection	Expert Syst Appl	10.1016/j.eswa.2019.04.039
47	Santini T et al. (2018)	PuRe: Robust pupil detection for real-time pervasive eye tracking	Comput Vis Image Underst	10.1016/j.cviu.2018.02.002
48	Saxena S et al. (2024)	Deep learning models for webcam eye tracking in online experiments	Behav Res Methods	10.3758/s13428-023-02190-6
49	Sevcenko N et al. (2022)	Theory-based approach for assessing cognitive load during time-critical resource-managing human–computer interactions: an eye-tracking study	J Multimodal User Interfaces	10.1007/s12193-022-00398-y
50	Shin H et al. (2024)	Evaluating and eliciting design requirements for an improved user experience in live-streaming commerce interfaces	Comput Hum Behav	10.1016/j.chb.2023.107990
51	Shojaeizadeh M et al. (2019)	Detecting task demand via an eye tracking machine learning system	Decis Support Syst	10.1016/j.dss.2018.10.012
52	Sievert A et al. (2018)	Reliability and Validity of Low Temporal Resolution Eye Tracking Systems in Cognitive Performance Tasks	Int J Mob Hum Comput Interact	10.4018/ijmhci.2018010103
53	Šoková B et al. (2024)	Fixation patterns in pairs of facial expressions-preferences of self-critical individuals	PeerJ Comput Sci	10.7717/peerj-cs.2413
54	Southwell R et al. (2022)	Gaze-based predictive models of deep reading comprehension	User Model User Adapt Interact	10.1007/s11257-022-09346-7
55	Sun N, Jiang Y (2025)	Eye movements and user emotional experience: a study in interface design	Front Psychol	10.3389/fpsyg.2025.1455177
56	Taieb-Maimon M et al. (2023)	Mining Eye-Tracking Data for Text Summarization	Int J Hum Comput Interact	10.1080/10447318.2023.2227827
57	Takemoto A et al. (2023)	Differentiating depression using facial expressions in a virtual avatar communication system	Front Digit Health	10.3389/fdgth.2023.1080023
58	Taylor P et al. (2015)	EyeFrame: real-time memory aid improves human multitasking via domain-general eye tracking procedures	Front ICT	10.3389/fict.2015.00017
59	Tuisku O et al. (2012)	Wireless Face Interface: Using voluntary gaze direction and facial muscle activations for human–computer interaction	Interact Comput	10.1016/j.intcom.2011.10.002
60	Vieira LN (2017)	How do measures of cognitive effort relate to each other? A multivariate analysis of post-editing process data	Mach Transl	10.1007/s10590-016-9188-5
61	Vojtechovska M et al. (2025)	Gaze controlled maps: scoping review of gaze-based interactions in geovisualisations	Int J Digit Earth	10.1080/17538947.2025.2510563
62	Wang H et al. (2024)	Expertise differences in cognitive interpreting: A meta-analysis of eye tracking studies across four decades	WIREs Cognitive Sci	10.1002/wcs.1667
63	Wibirama S et al. (2025)	Classification of Cognitive Load Using Deep Learning Based on Eye Movement Indices	IEEE Access	10.1109/ACCESS.2025.3613292
64	Wibirama S et al. (2019)	Physical discomfort and eye movements during arbitrary and optical flow-like motions in stereo 3D contents	Virtual Real	10.1007/s10055-019-00386-w
65	Yang L et al. (2025)	Cross-task cognitive workload estimation using eye tracking	Signal Image Video Process	10.1007/s11760-025-03931-0
66	Zemblys R et al. (2018)	Using machine learning to detect events in eye-tracking data	Behav Res Methods	10.3758/s13428-017-0860-3
67	Zhang L, Cui H (2022)	Reliability of MUSE 2 and Tobii Pro Nano at capturing mobile application users’ real-time cognitive workload changes	Front Neurosci	10.3389/fnins.2022.1011475
68	Zhang R et al. (2025)	Hierarchical intention recognition framework in intelligent human‒computer interactions for helicopter and drone collaborative wildfire rescue missions	Eng Appl Artif Intell	10.1016/j.engappai.2025.110037
69	Zhao S, Cheng S (2023)	Adaptive navigation assistance based on eye movement features in virtual reality	Virtual Real Intell Hardw	10.1016/j.vrih.2022.07.003
70	Zhu Y et al. (2024)	From Distraction to Action: Elevating Situation Awareness with Visual Assistance in Level 3 Autonomous Driving	Int J Hum Comput Interact	10.1080/10447318.2024.2350840

Appendix B

**Table 4:** Domain-level synthesis with representative studies (2012–2025)

Domain (n)	Representative studies	What eye tracking is used for in this domain	Recurrent limitations highlighted in the review
HCI / Human–Computer Interaction (n = 25)	Guerberof Arenas et al., 2021; Sevcenko et al., 2022; Castilla et al., 2023	Interface usability and user-state inference (e.g., workload, task demand, attention allocation); early ML-supported modeling of user states	Hardware variability and reporting heterogeneity; ecological validity constraints; dependence on calibration quality and drift control
Cognitive psychology (n = 20)	Southwell et al., 2022; Jang et al., 2013; Chiquet et al., 2020	Real-time indicators of attention, visual search, executive control and memory-related processing; task-driven inference on cognitive mechanisms	Predominance of lab-bound paradigms; task specificity and limited generalizability across settings/populations
Clinical psychological assessments (n = 18)	Cuve et al., 2022; Šoková et al., 2024; Modi & Singh, 2024	Exploratory characterization of atypical gaze/oculomotor patterns (ADHD/ASD and related profiles) and pupillary responses as complementary signals	Strong methodological heterogeneity; limited standardization; insufficient evidence for diagnostic validity / decision-making use; need for stronger validation
Emotional & affective research (n = 7)	Šoková et al., 2024; Modi & Singh, 2024	Emotion-related gaze allocation and pupillary responses as arousal indices; early attempts at classification using gaze features	High inter-individual variability; calibration sensitivity; comparability limited by different stimuli/protocols
