# Correction: Gaze and Eye-Tracking Perspectives for Psychological Research: A Narrative Review of Advances From 2012 to 2025

**DOI:** 10.7759/cureus.c426

**Published:** 2026-04-20

**Authors:** Maria Laura Mele, Stefano Federici

**Affiliations:** 1 Psychology, Myèsis Insight Center, Center for Research and Psychotherapy, Rome, ITA; 2 Psychology, Department of Philosophy, Social &amp; Human Sciences and Education, University of Perugia, Perugia, ITA

This article has been corrected to correct errors in the PRISMA diagram. The paragraph immediately preceding Figure 1 has also been revised to provide more detail on the literature search.

